# Under Pressure: Unmasking Idiopathic Intracranial Hypertension

**DOI:** 10.51894/001c.164587

**Published:** 2026-07-31

**Authors:** Michael Carter, Lauren Yangouyian, Ashley Yangouyian, Jared Tippett

**Affiliations:** 1 Henry Ford - Jackson

## 70

### Introduction

Headache is a common chief complaint in the emergency department. While most cases are benign, clinicians must remain alert to more serious causes. Idiopathic intracranial hypertension (IIH), or pseudotumor cerebri, is a rare disorder defined by elevated intracranial pressure without other identifiable causes. Because of the significant effects of increased intracranial pressure associated with IIH, prompt diagnosis is necessary to prevent permanent vision loss. Although lumbar puncture remains the gold standard for diagnosing IIH, point-of-care ultrasound measurement of the optic nerve sheath diameter is a rapidly growing tool that can aid in supporting the diagnosis of this disease.

### Case Description

A 32-year-old woman with a history of migraines presented to the ED per a referral from her optometrist, who noted bilateral optic disc edema. Further subjective data revealed two weeks of blurry vision primarily in the left eye, worsened with eye movement, and a new left sided migraine worse from her baseline. Neurologic examination revealed intact cranial nerves II-XII and no focal deficits. Visual acuity was 20/200 in both eyes. Initial imaging, including CT head, CT Venography, and CT angiography was performed and returned showing no acute intracranial abnormalities. Lumbar puncture revealed an opening pressure of 36 cm H₂O. Given our clinical suspicion of IIH, bedside ocular ultrasound demonstrated increased optic nerve sheath diameter, measuring 0.75 cm OS and 0.65 cm OD. The patient’s findings supported the diagnosis of IIH and was promptly started on acetazolamide 250 mg three times daily, with neurology and ophthalmology follow-up.

### Discussion

IIH is a significant diagnosis of exclusion that must be kept on clinicians’ radar when evaluating a headache. Diagnosis is supported by in-depth neuroimaging to rule out structural causes, lumbar puncture to evaluate opening pressure, and bedside optic nerve sheath diameter measurement. This case demonstrates the significance of noninvasive measures for detecting elevated intracranial pressure in the ED. Early recognition and treatment are essential to preserve vision and prevent long-term morbidity.

